# Analysis of apolipoprotein E genetic variation in patients with Alzheimer disease referred to Imam Reza Clinic, Rasht, Iran, in 2015

**Published:** 2017-10-07

**Authors:** Amir Reza Ghayeghran, Maryam Akbarshahi, Zivar Salehi, Ali Davoudi-Kiakalayeh

**Affiliations:** 1 Department of Neurology, School of Medicine, Guilan University of Medical Sciences, Rasht, Iran; 2 Department of Biology, School of Sciences, University of Guilan, Rasht, Iran; 3 Road Trauma Research Center, Guilan University of Medical Sciences, Rasht, Iran

**Keywords:** Alzheimer Disease, Apolipoprotein E, Genetic Variation

## Abstract

**Background:** Alzheimer disease (AD) is a progressive neurological degenerative disorder and the most common form of dementia. There are about 100 genes linked to AD including apolipoprotein E (ApoE). This gene exists in the form of three allele polymorphisms of ε_2_, ε_3_ and ε_4_ and six genotypes of ε_2_ε_3_, ε_2_ε_2_, ε_3_ε_3_, ε_2_ε_4_, ε_3_ε_4_, and ε_4_ε_4_. We aimed to study the association of ApoE polymorphism with AD in Guilan province, Iran.

**Methods:** The study group consisted of 70 AD patients and 100 healthy individuals as a control group. All subjects were recruited from 21 March to 22 September 2015 at Imam Reza Clinic, Rasht, Iran. The genomic deoxyribonucleic acid (DNA) was extracted from peripheral blood leucocytes, and subsequently, subjects were genotyped for ApoE using tetra-primer amplification refractory mutation system-polymerase chain reaction (ARMS-PCR). The association between the risk allele and AD was assessed using the MedCalc software.

**Results:** The distributions of ε_3_ε_3_, ε_3_ε_4_, ε_2_ε_2_, ε_2_ε_4_, ε_4_ε_4_ and ε_2_ε_3_ Genotypes among patients were 55.7%, 30.0%, 1.4%, 2.9%, 8.6%, 1.4% and in the controls were 79.0%, 8.0%, 0%, 1.0%, 1.0%, 11.0%, respectively. The genotype frequencies were significantly different between cases and the controls (P < 0.001). The individuals with the ε_4_ε_4_ and ε_3_ε_4_ genotypes had a greater risk for AD as compared to others; odds ratio (OR) = 12.15, 95% confidence interval (CI): 1.41-104.50, P = 0.020; OR = 5.32, 95% CI: 2.16-13.08, P = 0.003. In addition, the ε_4_ allele is significantly associated with higher AD risk among the studied population (OR = 5.63, 95% CI: 2.74-11.58, P < 0.001).

**Conclusion:** This case-control study suggests that the subjects with ε_4_ε_4_ and ε_3_ε_4_ genotypes had an increased risk for AD in Iranian population.

## Introduction

Alzheimer Disease (AD) is a progressive neurological degenerative disease and the most common form of dementia. It accounts for 50%-60% of dementia cases and affects quality of life in elderly people.^1^^-^^3^ According to the Alzheimer’s Disease International, it is estimated that there are currently 30 million people with dementia in the world which will increase to 100 million by 2050.^4^ It is also estimated that almost 13% of people over 65 years are affected, and its prevalence increases with age, so that 1% of people with 65 years old and younger, and 40% of persons aged over 90 years suffer from this disease.^5^ Less than 1% of all patients with AD experience early onset (before the age of 60-65 years) and 60% of the early AD is familial.^6^^,^^7^ It is proved that no environmental factors (e.g. head injury, viruses, toxins, lower education level) have a direct role in the pathogenesis of AD. Therefore, it seems that AD late onset results from unknown environmental factors on a predisposed genetic background.^8^^,^^9^

There are about 100 genes linked to AD including apolipoprotein E (ApoE), a risk factor for AD that has attracted much attention.^10^^,^^11^ ApoE gene, located on chromosome 19, is the genetic source of the most common form of AD with late onset. This gene is in the form of three alleles of ε_2_, ε_3_, and ε_4_, and six genotypes of ε_2_ε_3_, ε_2_ε_2_, ε_3_ε_3_, ε_2_ε_4_, ε_3_ε_4_, and ε_4_ε_4_.^12^ ApoE protein is expressed by all tissues, and is effective in the regulation of cell function of different tissues and organs in addition to lipid transfer.^13^ Human and animal studies clearly have shown that ApoE isoforms differentially affect the assembly and clean-up of β-amyloid. Evidence from genetic, pathologic and functional studies has shown that the imbalanced production and clearance of β-amyloid peptide in the brain leads to its accumulation, and eventually nerve degeneration and dementia.^2^^,^^6^ Many studies on genome have confirmed that ε_4_ allele of ApoE gene is the strongest genetic risk factor for AD. This allele is associated with an increased risk of both early and late AD. β-amyloid deposits in the form of senile plaques in ApoE ε_4_ carriers as compared to non-carriers.^14^ Therefore, ApoE genotypes strongly influence β-amyloid deposits in the form of senile plaques and lead to cerebral amyloid angiopathy.^15^ Clinical autopsy-based meta-analysis studies have shown that the risk of AD in individuals with one copy of ε_4_ allele (ε_3_ε_4_, ε_2_ε_4_) or two copies (ε_4_ε_4_) was higher among whites as compared to patients with genotype ε_3_ε_3._^16^ Although ε_3_ allele is the most common one, various studies have shown that ε_4_ allele in people with late family history and sporadic AD, in comparison with control group, has a higher frequency. ε_3_ allele has a moderate effect, and its impact on the disease pathology is a basic comparison for ε_4_ and ε_2_ isoforms due to a very high frequency. ε_2_ allele of the ApoE gene has a lower frequency and possesses protective effects against AD.^12^

In the view of the above-mentioned facts, the purpose of conducting this case-control study is to evaluate the association of ApoE polymorphism with the susceptibility to AD in Iranian population.

## Materials and Methods

The case-control study was conducted on 70 cases and 100 healthy controls. A questionnaire including information such as age, sex, family history of AD, and the race was used. All subjects were native Iranian living in the north of Iran, Guilan province. Patients’ mean age ± standard deviation (SD) was 77.1 ± 9.4, ranging from 65 to 89 years. Patients, diagnosed with AD, were recruited from 21 March to 22 September 2015, at Imam Reza Clinic of Guilan, Rasht. Identification and diagnosis of AD were performed based on National Institute of Neurological Disorders and Stroke-Alzheimer's Disease and Related Disorders Association (NINCDS-ADRDA) criteria. Patients diagnosed with Parkinson's disease or Parkinsonism at any time before the onset of dementia, patients with a history of stroke, history of alcohol abuse, or conclusive clinical history of schizophrenia or schizoaffective disorder before dementia onset were excluded from the study. Controls with the mean age of 74.7 ± 10.3 years (ranging from 65 to 87 years) were nonrelated and healthy individuals. Cases and controls were matched for age, and there were no significant differences between two groups (case and control) in terms of sex, race and family history of AD (P > 0.050). The characteristics of the cases and controls are shown in table 1. Informed consent for the genetic analysis was obtained from all participants. The study was conducted in accordance with the Declaration of Helsinki regarding the use of human samples.

For each sample, 1 ml blood was collected by venipuncture and drawn into Ethylenediaminetetraacetic acid (EDTA)-K3 coated tubes. Genomic deoxyribonucleic acid (DNA) was extracted from peripheral leukocytes using extraction kit following standard procedures. Extracted DNAs were frozen at -20 °C until the time of doing the molecular analysis.

**Table 1:** Characteristics of cases and controls

**Characteristics**		**Case (n = 70)**	**Control (n = 100)**	**P** *
Sex [n (%)]	Man	32 (45.7)	47 (47.0)	0.990
	Woman	38 (54.3)	53 (53.0)	
Family history of AD [n (%)]	Yes	19 (27.1)	23 (23.0)	0.660
	No	51 (72.9)	77 (77.0)	
Race [n (%)]	Gilak	51 (72.9)	74 (74.0)	0.810
	Talesh	9 (12.8)	14 (14.0)	
	Turk	6 (8.6)	5 (5.0)	
	Tat	4 (5.7)	7 (7.0)	

AD: Alzheimer disease

* Chi-square test

Genomic DNA quality was assessed by electrophoresis with 1% agarose gel. The gel was visualized by the gel documentation system.

The ApoE genotypes were determined by tetra-primer amplification-refractory mutation system-polymerase chain reaction (ARMS-PCR). The extracted DNA was used as a template for PCR. Amplifications were carried out using primers designed by Oligo software (version 7.54, Molecular Biology Insights Inc., Cascade, CO, USA). The reaction was performed for all samples after optimization of PCR conditions for amplification of the desired allele. PCR amplifications were carried out in a total volume of 25 µl containing 30 ng genomic DNA, 1x PCR buffer, 1.5 mM MgCl_2_, 0.2 mM deoxynucleotide triphosphate (dNTP), 0.5 mM each primer, and 1.5 U of superTaq DNA polymerase. At the end, PCR products were analyzed by agarose gel electrophoresis, and alleles and genotypes were identified based on the length of the used fragments and primers. 

The statistical significance of differences between groups was calculated by the chi-square test. A P of less than 0.050 was considered statistically significant. The odds ratios (OR) and 95% conﬁdence intervals (95% CI) were calculated using logistic regression to estimate the strength of the association between ApoE genetic variation and susceptibility to AD. All statistical analyses were conducted using the MedCalc software (version 12.1).

## Results

This case-control study included 70 patients with AD and 100 healthy controls. The distributions of ε_3_ε_3_, ε_3_ε_4_, ε_2_ε_2_, ε_2_ε_4_, ε_4_ε_4_ and ε_2_ε_3_ genotypes among patients were 55.7%, 30.0%, 1.4%, 2.9%, 8.6%, 1.4%, and in the controls were 79.0%, 8.0%, 0%, 1.0%, 1.0%, 11.0%, respectively. The genotype frequencies were significantly different between cases and the controls (P < 0.001). It was observed that the individuals with ε_4_ε_4_ and ε_3_ε_4_ genotypes had a greater risk of AD compared to others (OR = 12.15, 95% CI: 1.41-104.50, P = 0.020; OR = 5.32, 95% CI: 2.16-13.08, P = 0.003). The allele frequencies of ApoE were 71.4% ε_3_, 3.6% ε_2_ and 25.0% ε_4_ in the AD cases and 88.5% ε_3_, 6.0% ε_2_ and 5.5% ε_4_ in the controls. We observed a significant difference in allele distribution of ApoE between AD patients and the controls (P < 0.001). In addition, the ε_4_ allele is significantly associated with higher AD risk among the studied population (OR = 5.63, 95% CI: 2.74-11.58, P < 0.001) (Table 2).

**Table 2:** Genotype and allele frequencies of apolipoprotein E (ApoE) and its association with Alzheimer disease (AD)

**Genotype**	**Case [n (%)]**	**Control [n (%)]**	**OR (95% CI)**	**P** *
ε_3_ε_3_	39 (55.7)	79 (79.0)	1.00 (Ref)	-
ε_4_ε_4_	6 (8.6)	1 (1.0)	12.15 (1.41-104.50)	0.020
ε_3_ε_4_	21 (30.0)	8 (8.0)	5.32 (2.16-13.08)	0.003
ε_2_ε_2_	1 (1.4)	0 (0)	6.04 (0.24-151.62)	0.270
ε_2_ε_4_	2 (2.9)	1 (1.0)	4.05 (0.36-46.06)	0.260
ε_2_ε_3_	1 (1.4)	11 (11.0)	0.18 (0.02-1.48)	0.110
Allele				
ε_3_	100 (71.4)	177 (88.5)	1.00 (Ref)	-
ε_2_	5 (3.6)	12 (6.0)	0.74 (0.25-2.15)	0.580
ε_4_	35 (25.0)	11 (5.5)	5.63 (2.74-11.58)	< 0.001

OR: Odds ratio; CI: Confidence interval

*Chi-square test

## Discussion

Studies in human and transgenic mice have shown that brain β-amyloid levels and amyloid plaque loads are ApoE isoform-dependent, suggesting an important role of ApoE in modulating β-amyloid metabolism, aggregation, and deposition.^17^ ApoE gene, known to mediate the regulation of cholesterol and triglyceride metabolism, is immunochemically localized to the senile plaques, vascular amyloid, and neurofibrillary tangles of AD.^18^ Genome-wide association studies confirmed that ε4 allele of APOE is the strongest genetic risk factor for AD.^19^ To date, no study has investigated the association between the genotypes of ApoE and the AD risk in the Guilan province. The present study evaluated the effect of ApoE variation on AD in the north of Iran, Guilan. Our findings suggest that individuals with ε_4_ε_4_ genotype have the highest risks of developing AD. Moreover, the most frequent genotype was ε_3_ε_3_ in patients and controls in Guilan. Our analysis also confirmed a significant association of the ε_4_ allele with AD.

To date, many epidemiological studies have suggested a relationship between ApoE genetic variations and AD risk. In 2013, Sabbagh, et al. in their study showed that ApoE ε_4_ carriers had a significantly higher percentage of frequentscores for plaques and tangles in comparison with ApoE ε_4_ non-carriers for several brain regions.^12^ Furthermore, Altmann, et al. showed that APOE ε_4_ confers greater AD risk in women.^20^ Isbir, et al. in Turkey showed that there was a significantly higher frequency of the ApoE ε_4_ allele in the group of Alzheimer’s patients than in control subjects.^21^ In China, Zhou, et al. studied the relationship between ApoE gene polymorphism and AD in the case group and control group in Uyghurs and Han populations. The distinction was seen in both ethnic groups so that the frequency of ε_3_ε_4_ genotype and ε_4_ allele in case group of Uyghurs and Han were higher than those in the control group. ApoE ε_4_ allele was recognized as a risk factor for AD for both populations.^22^

Another study conducted by Gavett, et al. showed that more ε_2_ alleles were associated with less AD pathology and, in turn, with less severe dementia. In contrast, more ε_4_ alleles were associated with more pathology and more severe dementia.^23^ Mino, et al. showed that there was no statistically significant relationship between case group (with AD and dementia) and control group (without AD and dementia) in terms of sex and family history and distribution of ApoE alleles.^24^ A study conducted in 2014 reported that there is a statistically significant relationship between the types of ApoE and patients’ age so that risk alleles, such as ε_4_, decrease the age of onset as 3-6 months.^5^

ApoE ε_4_ allele frequency varies in different ethnic groups, and the mean has been estimated as 6.5 ± 13% in all groups. It has been reported that the lowest frequency was observed among Chinese and Japanese people (7.4 ± 0.8%), and the highest frequency was found among Sudanese (29%),^21^ and Finnish people (23-24%).^25^ Shahsavar, et al. also found that ε_3_ε_3_ genotype with a frequency of 48% was the most common genotype in their study population.^13^

Our study includes a small sample size, and statistically signiﬁcant results may occur by chance. It is also unwise to ignore other factors like environment and hereditary conditions that may predispose a person to AD, as there are other genes that may affect the susceptibility to AD. Thus, it will be necessary to assess the relationship between the genetic and environmental factors that influence the risks of AD in other studies.

## Conclusion

In conclusion, the results of this study provide further evidence that ε_4 _increases the risk of AD. However, a larger study that includes more samples may be necessary to confirm the findings.

## Notes:

**How to cite this article:** Ghayeghran AR, Akbarshahi M, Salehi Z, Davoudi-Kiakalayeh A. Analysis of apolipoprotein E genetic variation in patients with Alzheimer disease referred to Imam Reza Clinic, Rasht, Iran, in 2015. Iran J Neurol 2017; 16(4): 173-7.
